# What is the best method to assess the abdominal wall? Restoring strength does not mean functional recovery

**DOI:** 10.1590/0102-672020190001e1487

**Published:** 2020-06-26

**Authors:** André Vicente BIGOLIN, Renan Trevisan JOST, Rafaela FRANCESCHI, Rodolfo WERMANN, Rodrigo FALCÃO, Alexandre Severo DO-PINHO, Rodrigo Della Mea PLENTZ, Leandro Totti CAVAZZOLA

**Affiliations:** 1 Cirurgia do Aparelho Digestivo, Universidade Federal do Rio Grande do Sul, Porto Alegre, RS, Brasil; 2 Cirurgia do Aparelho Digestivo, Pontifícia Universidade Católica do Rio Grande do Sul, Porto Alegre, RS, Brasil; 3 Fisioterapia, Universidade Federal de Ciências da Saúde de Porto Alegre, Porto Alegre, RS, Brasil

**Keywords:** Hernia, Abdominal Wall, Electromyography, Muscle strength, Kinesiology, applied, Hérnia, Parede abdominal, Eletromiografia, Força muscular, Cinesiologia aplicada

## Abstract

**Background:**

Restoring the contractile function to the abdominal wall is a major goal in hernia repair. However, the core understanding is required when choosing the method for outcome assessment.

**Aim:**

To assess the role of the anterolateral abdominal muscles on abdominal wall function in patients undergoing hernia repair by analysis of correlation between the surface electromyography activation signal of these muscles and torque produced during validated strength tests.

**Methods:**

Activation of the rectus abdominis, external oblique, and internal oblique/transverse abdominis muscles was evaluated by surface electromyography during two validated tests: Step: 1-A, isometric contraction in dorsal decubitus; 1-B, isometric contraction in lateral decubitus; 2-A, isokinetic Biodex testing; and 2-B, isometric Biodex testing.

**Results:**

Twenty healthy volunteers were evaluated. The linear correlation coefficient between root mean square/peak data obtained from surface electromyography signal analysis for each muscle and the peak torque variable was always <0.2 and statistically non-significant (p<0.05). The agonist/antagonist ratio showed a positive, significant, weak-to-moderate correlation in the external oblique (Peak, p=0.027; root mean square, 0.564). Surface electromyography results correlated positively among different abdominal contraction protocols, as well as with a daily physical activity questionnaire.

**Conclusions:**

There was no correlation between surface electromyography examination of the anterolateral abdominal wall muscles and torque measured by a validated instrument, except in a variable that does not directly represent torque generation.

## Graphical Abstract

[Fig f1a]

**Figure f1a:**
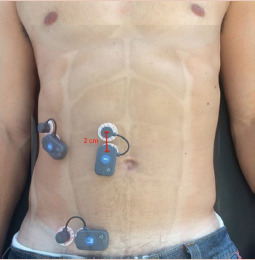
Placement of electrodes for wireless surface electromyography (SEMG) of the rectus abdominis, external oblique, and transversus/internal oblique muscles. A distance of 2 cm between centers was maintained

## RESUMO

**Racional:**

A devolução da funcionalidade contrátil da parede abdominal é uma das metas no reparo das hérnias abdominais. Contudo, o entendimento do core deve necessariamente fazer parte na escolha do método de avaliação desse desfecho.

**Objetivo:**

Avaliar o papel dos músculos da parede anterolateral na função da parede abdominal com base na correlação entre o sinal de ativação muscular obtido na eletromiografia de superfície e torque produzido durante testes de força validados.

**Métodos:**

A ativação dos músculos reto abdominal, oblíquo externo, e oblíquo interno e transverso foi avaliada por eletromiografia de superfície durante dois testes validados. Etapa: 1-A, contração isométrica em decúbito dorsal; 1-B, contração isométrica em decúbito lateral; 2-A, teste isocinético no Biodex; e 2-B, teste isométrico no Biodex.

**Resultados:**

Foram avaliados 20 voluntários saudáveis. O coeficiente de correlação linear entre os dados de valor quadrático médio/Pico obtidos análise do sinal da eletromiografia de superfície para cada músculo e o Pico de torque foram sempre <0,2 e estatisticamente insignificantes (p<0.05). A relação agonista/antagonista demonstrou correlação positiva, significativa e de fraca a moderada no músculo externo oblíquo (Pico, p=0,027; valor quadrático médio, 0,564). Os resultados eletromiografia de superfície estiveram positivamente correlacionados nos diferentes protocolos de contração abdominal e também com um questionário de atividade física diária.

**Conclusões:**

Não houve correlação entre o exame de eletromiografia de superfície e o torque mensurado por um instrumento validado, exceto em uma variável que não representa diretamente a geração de torque.

## Graphical Abstract

**Figure:** Colocação dos eletrodos para eletromiografia de superfície sem fio (EMGs) dos músculos reto abdominal, oblíquo externo e transverso/oblíquo interno. Foi mantida distância de 2 cm entre os centros

## INTRODUCTION

The anterolateral abdominal wall consists of the rectus abdominis muscle, the internal and external obliques, and the transverse abdominis and its aponeuroses. These muscles play a key role among the 26 other pairs that make up the core. This muscular system supports the abdominal and lumbopelvic regions, stabilizing the spine and pelvis and maintaining kinetic organization during functional movement. When functioning properly, it promotes not only strength but stability^12^ ^,^ ^23^.

Any structural or neurological damage to this muscular-aponeurotic system can impair quality of life. These effects are clear in patients with incisional hernia^16^ ^,^ ^29^.

In the United States, 3.2 billion dollars were expended on ventral hernia treatment in 2006 alone^22^. In France, these costs were estimated at approximately 84 million euros in 2011^29^.

However, the degree to which muscle function can be reestablished and the extent to which this is clinically relevant to a patient’s daily life is still under debate. Since recurrence has been significantly reduced with the advent of mesh repair^7^, the focus has shifted to new quality-of-life outcomes. In 2011, a group of researchers validated the Biodex Multi-Joint System 4 Pro electronic dynamometer^6^. With this instrument, kinetics is controlled (isokinetic) and abdominal contraction force is translated into torque (in Newtons per second).

Surface electromyography (SEMG), also called kinetic electromyography due to its ability to evaluate muscle activation during movement, is a commonly used instrument in training and rehabilitation analysis of the core muscles and for patients with low back pain^2^ ^,^ ^14^ ^,^ ^17^. In a review of the literature, 87 studies involving SEMG and abdominal-wall muscles were found between 1950 and 2008^15^. Variability between tests, poor technical descriptions, small sample sizes, no description of the physical activity level of the evaluated individuals, and non-standardized signal capture and processing techniques were some of the problems observed.

Within this context, the objective of the present study was to determine standardized activation signal values for the muscles of the anterolateral abdominal wall during isometric and isokinetic exercises, validate these in Biodex, and correlate these results with torque data obtained during performance of the aforementioned exercises.

## METHODS

The study was approved by the institutional ethics committee under protocol number 928582, and conducted in accordance with the provisions of the Helsinki Declaration. All volunteers provided written informed consent prior to inclusion in the study. Additional informed consent was obtained from all individual participants for whom identifying information is included in this article.

### Study protocol

Cross-sectional study of healthy volunteers. The volunteers were interviewed to assess for eligibility. The main exclusion criterion was history of any incision or hernia in the abdomen or groin region. Presence of comorbidities, ASA classification >1, body mass index (BMI) >30, age >50 or <18 years, any orthopedic condition that causes functional impairment, and comorbidity severity score =2 were the other exclusion criteria.

All volunteers had his waist circumference, weight, and height measured. Two validated questionnaires were applied. First, the International Physical Activity Questionnaires (IPAQ), which assesses walking, moderate and vigorous activities in four domains (work, transport, domestic and gardening, and leisure), asked independently^3^. The results are expressed in MET-minutes/week, calculated using a mathematical formula and the SF-36, which measures individual quality of life in eight physical and mental domains. Results are expressed as a score of 0 to 100^5^.

**FIGURE 1:**
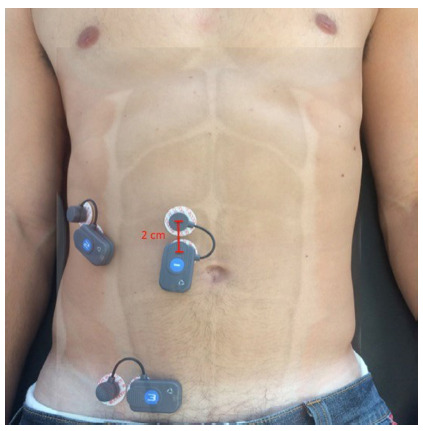
Placement of electrodes for wireless surface electromyography (SEMG) of the rectus abdominis, external oblique, and transversus/internal oblique muscles. A distance of 2 cm between centers was maintained

For SEMG, the skin was epilated, exfoliated, and wiped with alcohol. Electrodes were then attached with a center-to-center spacing of 2 cm. The rectus abdominis (RA), external oblique (EO), and internal oblique, the latter together with the transverse abdominis (IO/TA), were evaluated. Figure 1 shows the electrode positioning for each muscle. The positioning of abdominal electrodes for the RA and EO was as described by Ng et al.^16^. This position follows the orientation of muscle fibers, ensuring low crosstalk between the EO and IO/TA^1^.

All data were collected using a BTS FREEEMG 1000 system with a sampling rate of 1.0 kHz, which includes super-lightweight (10 g) wireless electrodes with a maximum transfer distance of 20 m, and analyzed in Smart Analyzer software (v. 1.10.465.0). The raw signals were filtered to a bandwidth of 20-500 Hz, and the data thus obtained were analyzed. Peak activation and root mean square (RMS) were calculated. The results were normalized on the basis of maximum voluntary contraction (MVC). A 120-second break was given between sets to avoid fatigue. Abdominal contraction was evaluated in two steps:

### Step 1 - Isometric tests on a backboard

####  Exercise 1-A 

**FIGURE 2:**
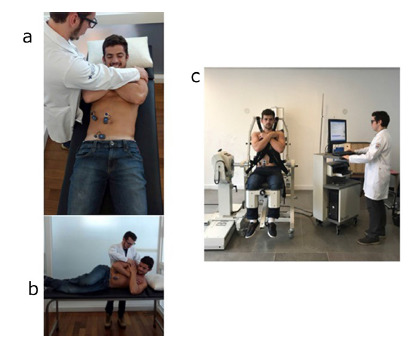
A) Isometric contraction against the examiner’s resistance in the supine position; B) isometric contraction against the examiner’s resistance in the lateral position; C) proper positioning in the Biodex device

With the spine straight, the volunteer flexed from the hip and knee while supine on a backboard. Against the examiner’s resistance, three sets of maximal contractions were sustained for 5 s (Figure 2).

####  Exercise 1-B 

In the lateral position, with the spine flexed and lateral trunk rotation, the volunteer performed three sets of maximal contractions, sustaining them for 5 s (Figure 2).

### Step 2

A Biodex Multi-Joint System 4 Pro isokinetic dynamometer (Biodex Corporation, Shirley, NY, USA), which can work on specific muscle groups in isokinetic and isometric tests, was used for this step. The system was attached to the dorsal muscles to evaluate contraction force. The patient was positioned in the module with the thigh, hip, and trunk immobilized, feet supported and at a 90° angle between the femur and the hip, measured at the iliac crest with a goniometer (Figure 2).

####  Exercise 2-A 

Three sets of five isokinetic contractions at an angular velocity of 60°/sec were performed. The range of motion was 40° to 80° to 110°.

####  Exercise 2-B 

Three sets of 5-second maximal isometric contractions were performed against machine resistance at an 80° angle.

### Statistical analysis

Means and standard deviations, as well as quartiles, minimums, and maximums, are presented for each variable. The data were analyzed in PASW Statistics for Windows, Version 18.0, with a significance level of 0.05 for all analyses. To compare SEMG results between different muscles and exercises, a generalized estimating equations model was used. For this comparison, an exchangeable correlation matrix, a robust estimator covariance matrix, and normal distribution with identity function were used, as well as post-hoc Bonferroni multiple comparisons. For a 90% chance of detection with a 5% significance level and an increase in the assessed outcome from 52 to 71^4^ ^,^ ^11^, the sample size was calculated as 20 patients.

## RESULTS

Twenty volunteers (10 men and 10 women) were evaluated. The mean (SD) age was 26 years (23-34), and the mean BMI was 22.7 kg/m² (minimum 18, i.e., underweight; maximum 27.5, i.e., overweight), which was within ideal limits, although the interquartile range for most of the sample population was within either normal or ideal limits. No obese individuals were evaluated. The mean waist circumference was 70.9 cm (Table 1).

**TABLE 1:** Anthropometric profile of the study population

Variable	Mean (SD)	IQR	Range (min-max)	n
Weight (kg)	66.0 (13.9)	68.5 (52.0; 77.0)	47.0 - 91.0	20
Height (cm)	169.0 (10.6)	167.0 (159.0; 176.0)	155.0 - 187.0	20
AWC (cm)	70.9 (20.2)	78.5 (67.5; 83.0)	26.0 - 90.0	20
BMI (kg/m²)	22.7 (3.0)	22.3 (20.1; 25.5)	18.0 - 27.4	20

AWC=abdominal wall circumference; SD=standard deviation; IQR=interquartile range; BMI=body mass index

Table 2 describes the normalized results resulting from analysis of muscle signal during the performance of isokinetic tasks in the Biodex system.

**TABLE 2:** Normalized SEMG data obtained from analysis of the rectus abdominis, external oblique, and transverse abdominis/internal oblique during standardized isometric and isokinetic exercises

Step 3, Exercise A: Isokinetic Biodex					
SEMG data	Muscle	Mean (SD)	IQR	Range (min-max)	n
Peak	RA	72.7 (18.6)	73.3 (65.7; 83.6)	83.6 - 30.4	20
	EO	73.7 (17.2)	77.8 (60.1; 85.9)	85.9 - 32.7	20
	TA/IO	75.9 (16.5)	77.8 (61.5; 90.3)	90.3 - 45.6	16
RMS	RR	49.7 (17.1)	49.2 (42.1; 59.3)	59.3 - 12.6	20
	EO	50.3 (18.6)	44.8 (41.3; 68.9)	68.9 - 22.8	20
	TA/IO	46.8 (16.1)	44.2 (37.2; 54.6)	54.6 - 16.1	16
Step 3, Exercise B: Isometric Biodex					
SEMG data	Muscle	Mean (SD)	IQR	Range (min-max)	n
Peak	RA	59.3 (22.5)	60.4 (40.6; 76.8)	76.8 - 15.4	20
	EO	59.8 (19.1)	53.8 (46.7; 67.9)	67.9 - 27.9	20
	TA/IO	61.7 (19.8)	65.7 (42.0; 74.1)	74.1 - 31.4	16
RMS	RR	52.0 (21.7)	55.7 (33.1; 67.8)	67.8 - 12.5	20
	EO	55.7 (21.5)	52.4 (40.3; 68.3)	68.3 - 26.3	20
	TA/IO	50.2 (19.3)	46.6 (37.3; 63.7)	63.7 - 20.3	16

RA=rectus abdominis; EO=external oblique; TA/IO=transverse abdominis and internal oblique; SD=standard deviation; IQR=interquartile range; n=number of subjects; RMS=root mean square

### Correlation tests

####  Between methods of functional assessment (SEMG vs.Biodex, IPAQ) 

**FIGURE 3:**
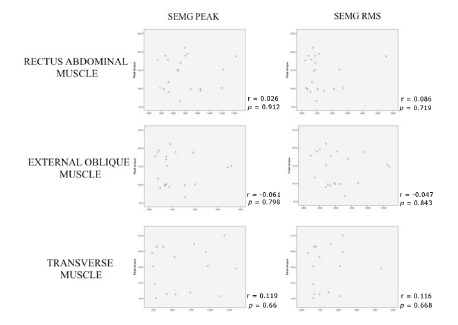
Correlation between Peak and RMS signal obtained by electromyography and Peak torque results obtained in the Biodex isokinetic test

**FIGURE 4:**
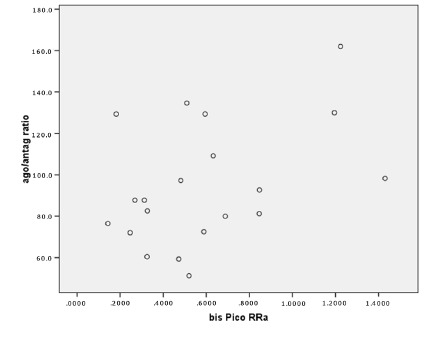
Correlation of the agonist/antagonist ratio obtained from the Biodex test with Peak signal of the rectus abdominis muscle obtained by SEMG during isokinetic exercise r=0.421; p=0.064.

When normalized SEMG results were correlated with the result of Biodex isokinetic and isometric tests (peak torque), the correlations found were weak and non-significant, and variably positive or negative. The only exception to this rule was agonist/antagonist data, which showed a weak but always positive, and sometimes significant correlation (Figures 3 and 4, Table 3).

**TABLE 3:** Linear correlations between normalized SEMG data and peak torque and agonist/antagonist ratio values obtained on Biodex testing

	Isometric test		Isokinetic test	
SEMG	Peak torque	Agonist/antagonist ratio	Peak torque	Agonist/antagonist ratio
Rectus muscle Peak	0.026 (0.912) [20]	0.421 (0.064) [20]	-0.176 (0.459) [20]	0.405 (0.077) [20]
Rectus muscle RMS	0.086 (0.719) [20]	0.289 (0.217) [20]	0.015 (0.949) [20]	0.240 (0.308) [20]
Transversus/internal oblique RMS	0.116 (0.668) [16]	0.309 (0.244) [16]	0.176 (0.515) [16]	0.131 (0.630) [16]
Transversus/internal oblique Peak	0.119 (0.660) [16]	0.348 (0.187) [16]	0.193 (0.473) [16]	0.238 (0.374) [16]
External oblique Peak	-0.061 (0.798) [20]	0.493 (0.027) [20]*	0.113 (0.636) [20]	0.312 (0.181) [20]
External oblique RMS	-0.047 (0.843) [20]	0.564 (0.010) [20]*	0.039 (0.872) [20]	0.425 (0.062) [20]

SEMG=surface electromyography; r=Pearson correlation coefficient (p) [n]; *p <0.05

The correlation of SEMG results with waist circumference was always negative and often strong and statistically significant. BMI had a similar negative correlation, but with lower magnitude and significance. The correlation with weight was variable, but predominantly negative. The correlation with height was predominantly positive, but did not reach statistical significance.

When IPAQ total scores were correlated with SEMG data, there was a trend toward positive correlation. Significant correlations were found between RA Peak and RMS values with total walking time (MET-minutes/week) in all of Step 1. During Step 2, the results were positive, but without statistical significance. This may be attributable to the small sample size.

####  Between different abdominal contraction exercises 

**FIGURE 5:**
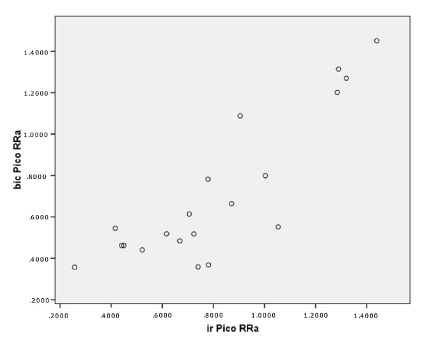
Correlation between SEMG findings obtained through analysis of the rectus abdominis muscle during isokinetic (Biodex) and isometric (forearm plank) exercise. r=0.863; p<0.001

There was always a strong, significant, and positive correlation between SEMG scores for both Peak and RMS when Step 1 exercise scores were correlated with Step 2 scores for RA, EO, and IO/TA (Figure 5).

As normalization was performed specifically for each muscle and each exercise on the basis of MVC, no attempts were made to assess correlation between different muscles and different tests.

## DISCUSSION

An understanding of the contractile dynamics of the lumbar, abdominal, and pelvic muscles and their interaction as a single unit when performing movements and exerting force provided the theoretical framework for the concept of the “core” in anatomy and physiology. These concepts of kinesiology have already been applied to the study of several conditions whose pathogenesis is directly or indirectly associated with dysfunction of the muscles that make up this complex system^2^ ^,^ ^13^ ^,^ ^17^ ^,^ ^28^. 

In an attempt to allow more in-depth research into the etiologies of low back pain, the results of isokinetic work have been correlated with electrophysiological findings in several previous studies^13^ ^,^ ^14^ ^,^ ^17^ ^,^ ^21^. 

However, only recently has knowledge from kinesiology begun to be considered and applied to the study of pathological conditions which affect the anterolateral abdominal wall directly. Several instruments purported to assess abdominal wall function have been evaluated. Abdominal-wall hernias have a major impact on patient quality of life. As advances in hernia repair have led to better clinical outcomes, research focus has shifted toward functional outcomes, focusing particularly on quality-of-life issues**.**

In 2011, Parker et al. ^20^ developed a pilot study aiming to develop a clinical protocol to assess abdominal wall strength in patients with abdominal wall hernias. In this study, they tested the reliability and reproducibility of a three-step protocol involving functional movements. However, the results of their tests were dependent on clinician evaluation. The use of machines has made such analysis less subjective.

The Biodex system was validated in patients with abdominal wall hernia based primarily on its correlation with IPAQ results^6^. Johansson et al. ^10^ and den Hartog et al. ^4^ have used this protocol in patients after hernia repair. The former found no significant differences in terms of functional gain when comparing three techniques of open hernia repair. The latter evaluated patients later in the postoperative period and found greater abdominal-wall strength in those who had undergone open ventral hernia repair compared to those who had undergone laparoscopic repair. The main hypothesis suggested was that the laparoscopic technique did not provide complete closure of hernia defects during surgical repair.

Nevertheless, Shestak et al. ^26^ were the only authors to compare patients in the pre- and postoperative periods. They used the Cybex dynamometer and found an increase in force generation after hernia repair.

There is still a lack of evidence regarding abdominal wall function in patients with hernia. Only seven studies were found in the most recent literature review at the time of writing^9^.

Our study is the first to use electromyography in a validated isokinetic test protocol for patients with abdominal wall hernia. However, there was no significant correlation between the muscle activation signal results obtained in SEMG with those of isometric and isokinetic tests.

The level of activation is not an independent factor for torque production during muscle contraction, i.e., maximum force production during the task under investigation does not necessarily produce a maximum activation level, and vice-versa. Other factors are expected to play an important role.

Some studies^24^ ^,^ ^25^ have already shown correlation with EMG findings and strength measurements. On the other hand, Pope et al.^21^ and McGill et al. ^14^ also failed to find an always linear correlation between kinetic activity and torque. They also identified a significant interaction of agonist/antagonist activation, which leads one to believe that, during some movements, the role of the muscle is much more a stabilizing one than a torque-generating one*.* Kumar et al.^13^ showed only weak correlations between SEMG findings and strength in isokinetic and isometric activation activities. They also found an interesting paradoxical relationship involving torque generation and muscle activation signal. Increasing the speed of movement from an isokinetic contraction led to a decrease in torque and increase in SEMG signal. More muscle activation is expended to ensure stability and deform the ligament structures that (according to their elasticity) restrict the production of movement, thus decreasing torque production. An increasing velocity of motion can be a risk factor for ligament injury if it exceeds the capacity of the safety mechanism provided by muscle activation. These data lead to the hypothesis that, if there is no stability in how movements are performed, the increase in strength that follows hernia repair can cause or perpetuate musculoskeletal injury and, consequently, pain.

Stability is the ability of the body to control the whole range of motion of a joint so there is no major deformity, neurological deficit, or incapacitating pain^19^. The kinematic response of the trunk muscles is proportional to the stability of the spine. In one study, electromyography was used to evaluate different instability devices used to train core muscles^27^. In 20 patients, significant differences in muscle activation were found between five exercises. The only correlation found in our study involved the unique torque data related to inter-muscle cooperation and stability: the agonist/antagonist ratio.

In an attempt to achieve more functional surgical repairs, some authors advocate closure of the linea alba in the treatment of ventral hernias. Sometimes, separation of components of the abdominal wall is necessary to ensure a more tension-free repair^2^. Recently, the transversus abdominis release (TAR) technique has shown good outcomes and lower morbidity in the long term. In this technique, the major concern is with the flap donor site, where transversus muscle injury occurs^18^.

The transversus abdominis muscle plays a fundamental role in trunk stability.This muscle is activated or “pre-activated” 30-100 ms before the first contraction results^8^. It is an essential core mechanism to protect the vertebrae and joints from injury during movement generation. 

Nevertheless, the TAR technique does not seem to impair stability, promoting improvement of low back pain and quality of life during the first six months postoperatively. In this study, stability results could be confronted with muscle activation data to ensure that the outcome of the performed exercise was not simply ensured by the compensatory activity of agonist and antagonist muscles^7^.

A more in-depth understanding of the mechanics of abdominal wall contraction is essential for better treatment of patients with abdominal hernias, as orthopedics did for the knee and spine. A good kinematic outcome is key for physical activity and, consequently, quality of life.

## CONCLUSION

The present study provides the basis for electromyographic evaluation of the muscles involved in contraction of the anterolateral abdominal wall after complex abdominal reconstruction and repair by different techniques. It is suggested that such evaluation should be performed in parallel to strength assessment so that continuous, normalizable, and comparable variables can be obtained for use in functional evaluation. The positive correlation between muscle activation tests and other functional evaluation instruments, such as the SF-36 and IPAQ, suggest that SEMG is a valid and feasible method.
